# Potential Causal Association between Elevated Gamma-Glutamyl Transferase Level and Stroke: A Two-Sample Mendelian Randomization Study

**DOI:** 10.3390/biom13111592

**Published:** 2023-10-29

**Authors:** Young Lee, Je Hyun Seo

**Affiliations:** 1 Veterans Medical Research Institute, Veterans Health Service Medical Center, Seoul 05368, Republic of Korea; lyou7688@gmail.com; 2 Department of Applied Statistics, Chung-Ang University, Seoul 06974, Republic of Korea

**Keywords:** stroke, mendelian randomization, gamma-glutamyl transferase, single-nucleotide polymorphisms, alcohol, atrial fibrillation, body mass index

## Abstract

Researchers have suggested a potential relationship between gamma-glutamyl transferase (GGT) level and stroke. We investigated a potential causal relationship between GGT level as exposures and stroke and stroke subtypes (cardioembolic, small vessel, and large artery) in a European population. We performed a two-sample Mendelian randomization (MR) study using the genome-wide association study (GWAS) data from the UK Biobank as the exposure set. For the outcome set, we used stroke in the GWAS data from the GIGASTROKE Consortium. We considered alcohol consumption, atrial fibrillation, and body mass index as confounders. We used PhenoScanner searches for removal of SNPs and multivariable MR analysis for assessing confounders. We observed significant causal associations between GGT level and stroke (odds ratio [OR] = 1.23, 95% CI = [1.05–1.44], and *p* = 0.012 with IVW; OR = 1.19, 95% CI= [1.02–1.39], and *p* = 0.031 with MR-PRESSO). These results were consistent after removing SNPs related to confounding factors. Similarly, in multivariable MR, GGT was associated with stroke after adjusting for confounding factors (OR = 1.30, 95% CI 1.07–1.60), *p* = 0.010). Because GGT level has a causal relationship with stroke, researchers should test its significance as a potential risk factor for stroke. Additional research is required to validate these results.

## 1. Introduction

Stroke is a leading cause of death and accounts for the largest proportion of neurological disorders that are more often disabling than fatal; it causes both physical and mental disability [1,2,3]. With the growing burden of stroke, novel prevention strategies that target modifiable risk factors are needed. Cardiovascular disease (CVD) risk factors are well known to be independent risk factors for stroke [4,5], but other risk factors for strokes should also be considered.

Recently, researchers have shown interest in the potential role of liver function in the development of CVDs [6]. For example, the gamma-glutamyl transferase (GGT) level is an indicator of hepatobiliary dysfunction and alcohol abuse. GGT is located on the cellular membrane and is responsible for regulating the entry of amino acids and peptides into the cell in the form of γ-glutamyl peptides [7]. It is also involved in maintaining the physiological concentration of glutathione in cells and reflects the oxidation–antioxidant balance in the body [8,9]. According to previous studies, GGT level is associated with a CVD diagnosis [9,10,11]. After a period of observation, researchers found that individuals with a higher GGT level were more likely than those with a lower GGT level to experience stroke [12,13]. Researchers also investigated the relationship between GGT level and adverse CVD clinical outcomes in stroke patients [14,15]. In addition, a previous study reported that an elevated GGT level is related to stroke recurrence and transient ischemic attack [13]. However, after adjusting for multiple factors, a previous study found that the association between GGT activity and stroke was no longer significant, suggesting that GGT activity might not be a good predictor of the severity of cardio-cerebrovascular diseases [16]. Therefore, whether GGT level has a causal association with stroke remains to be elucidated.

Mendelian randomization (MR) is a genetic epidemiological technique that uses genetic variants associated with potential exposures as instrumental variables (IVs) to evaluate their causal effects on disease outcomes [17,18]. Several previous studies have used MR analyses to find risk factors for stroke [19,20,21,22]. An MR study has shown an association between high level of bilirubin and decreased stroke risk in a Korean population, in agreement with observational results [23], whereas non-alcoholic fatty liver disease has a causal association with small vessel occlusion [6]. As previously described, increased circulating GGT activity is an indicator of insufficient antioxidant levels and increased oxidative stress and indicates a heightened inflammatory state in vivo. The inclusion of several confounding factors in the study of GGT and stroke, alongside the implementation of MR analysis and multivariable MR, are anticipated to yield more robust and reliable research findings. The investigation incorporated multiple factors such as alcohol intake [24], atrial fibrillation [25], and body mass index [26] as confounders. In this study, we investigated the causal effects of GGT level on stroke and stroke subtype in a two-sample MR analysis that used summary statistics from the UK Biobank (UKB) [27] as the exposures and summary statistics from the Stroke Consortium (GIGASTROKE Consortium) dataset as the outcomes [28]. Furthermore, to mitigate the impact of allele frequency analysis across racial groups, an analysis was conducted using data solely from a European population.

## 2. Materials and Methods

### 2.1. Study Design

The Institutional Review Board of the Veterans Health Service Medical Center approved this study protocol (IRB No. 2023-03-004) and waived the need for informed consent because of its retrospective design. This study was conducted in compliance with the tenets of the Helsinki Declaration.

### 2.2. Data Sources

**Figure 1:**
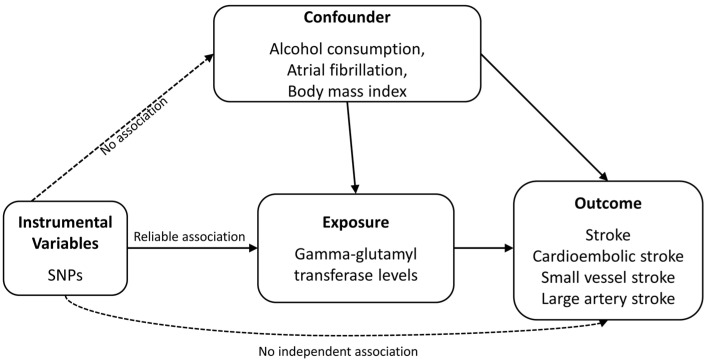
Diagram of two-sample Mendelian randomization analysis. SNP, single-nucleotide polymorphism.

Figure 1 is a schematic of the analytical study design. To investigate the causal effects of GGT level on the risk of stroke and stroke subtypes, we selected the following datasets. (1) As exposure data, we used the summary statistics from the UKB genome-wide association study (GWAS) (*n* = 437,194 for GGT) [27]. (2) As outcome data, we used the summary statistics from the stroke GWAS (2,036,031 (136,047 cases + 1,899,984 controls)) and stroke subtype GWAS [1,245,612 (10,804 cases + 1,234,808 controls) for cardioembolic stroke, 1,241,619 (6811 cases + 1,234,808 controls) for small vessel stroke, 1,241,207 (6399 cases + 1,234,808 controls) for large artery stroke] of European samples from the GIGASTROKE Consortium [28]. In addition, alcohol consumption, atrial fibrillation, and body mass index were considered as confounding factors. Table 1 describes the datasets whose summary statistics we used.

### 2.3. Selection of the Genetic Instrumental Variables

Single-nucleotide polymorphisms (SNPs) associated with GGT at the GWAS threshold (*p* < 5.0 × $10^{-8}$) were extracted. We pruned theses SNPs by linkage disequilibrium (LD; r^2^ = 0.001, clumping distance = 10,000 kb) to ensure that each IV was independent from the others. The linkage disequilibrium between the SNPs was calculated using the 1000 Genome Phase III European data as a reference. The MR analysis was conducted using the selected SNPs as IVs. We also checked the PhenoScanner GWAS database (http://phenoscanner.medschl.cam.ac.uk (accessed on 7 July 2023)) for each SNP and its proxy (LD: r^2^ > 0.8) to determine whether it was associated with alcohol consumption, atrial fibrillation, or body mass index (*p* < 1.0 × $10^{-5}$) as confounding factors [24,25,26,32,33]. We conducted an additional MR analysis after eliminating confounding factor-related SNPs. We used the 1000 Genomes Phase III dataset (European population) as the reference panel to compute the LD. We assessed the F-statistics for each individual genetic instrument to ensure the reliability of the method. F-value > 10 indicated that the causal estimation was unlikely to be biased due to weak instruments [34].

### 2.4. Mendelian Randomization

The MR analysis was conducted based on the following assumptions for the IVs: (1) should demonstrate a substantial relationship with the exposure; (2) should have no connection to confounders of the exposure–outcome relationship; and (3) should influence the outcomes only through exposure, with no directional horizontal pleiotropy effect. As the primary analysis method, we used inverse variance-weighted (IVW) MR with multiplicative random effects [34,35,36]. Additionally, we used the weighted median [37], MR-Egger (with and without adjustment via the simulation extrapolation [SIMEX] method) regression [38,39], and MR polyhedral sum of residuals and outliers (MR-PRESSO) [40]. As a sensitivity analysis, we calculated MR estimates while excluding SNPs associated with confounding factors (alcohol consumption, atrial fibrillation, and body mass index). The IVW method is most effective when all genetic variations satisfy the three assumptions for IVs listed above [41]. If one or more of the variants is invalid, the IVW estimate can be biased [37]. The weighted median approach produces accurate estimates of causality even if 50% of the instruments are incorrect [37]. The MR-Egger technique allows estimation of appropriate causal effects even in the presence of pleiotropic effects, permitting a non-zero intercept that clearly demonstrates the average horizontal pleiotropic effects [38]. The MR-Egger with SIMEX can be used to rectify the bias when no measurement error assumption is broken [39]. The MR-PRESSO test, which identifies outliers, adjusts the results of the IVW analysis for horizontal pleiotropy by deleting the outliers [40]. Heterogeneity for IVW and MR-Egger was evaluated using Cochran’s Q and Rücker’s Q’ statistics, respectively [35,42]. We assessed directional horizontal pleiotropy using the MR-PRESSO global test. Therefore, we interpret the results according to the appropriate MR analysis method [43]. *p*-value < 0.05 for Cochran’s Q statistic, Rücker’s Q’ statistic, and the MR-PRESSO global test indicate possible pleiotropy in the genetic variations. We also performed multivariable IVW MR analysis to assess confounders. All analyses were performed using the TwoSampleMR and simex packages in R version 3.6.3 (R Core Team, Vienna, Austria).

## 3. Results

### 3.1. Heterogeneity and Horizontal Pleiotropy of the Instrumental Variables

Among the 16,901,631 genetic variants in the exposure GWAS data, 68,949 were significant for GGT (*p* < 5.0 × $10^{-8}$), and the IVs finally selected after combining each outcome GWAS dataset and clumping numbered 321 for stroke, 319 for cardioembolic stroke, 315 for small vessel stroke, and 317 for large artery stroke. After removal of confounding factor-related SNPs, there were 272 IVs for stroke, 271 for cardioembolic stroke, 268 for small vessel stroke, and 268 for large artery stroke. The F values for all the SNPs selected as IVs are larger than 10, indicating a low probability of weak instrument bias, and the mean F values are greater than 130 (Table 2 and Supplementary Table S1). The premise of no measurement error was not broken in any of the outcomes (I^2^ > 90 in Table 2). Supplementary Table S1 contains detailed information about each IV, such as whether it is an MR-PRESSO outlier and whether it is known to be related to confounding factors. A pleiotropic effect was observed in stroke, small vessel stroke, and large artery stroke through the Cochran’s Q test (*p* < 0.05) from IVW, Rücker’s Q′ test (*p* < 0.05) from MR-Egger, and from the MR-PRESSO global test (*p* < 0.05) before removing SNPs related to confounding factors (Table 2). Therefore, the MR-PRESSO results were regarded as the key outcomes [43]. When confounding factor-related SNPs were eliminated, IV for large artery stroke satisfied assumptions (Q, *p* > 0.05; Q’, *p* > 0.05; MR-PRESSO global test, *p* > 0.05), at which point IVW was recommended (Table 2) [43]. For cardioembolic stroke, the IVs satisfied the assumptions (Q, *p* > 0.05; Q’, *p* > 0.05; MR-PRESSO global test, *p* > 0.05) regardless of whether confounding factor-related SNPs are removed, and IVW was recommended for MR analyses (Table 2) [43].

### 3.2. Mendelian Randomization for the Causal Association between Gamma-Glutamyl Transferase and Stroke

**Figure 2:**
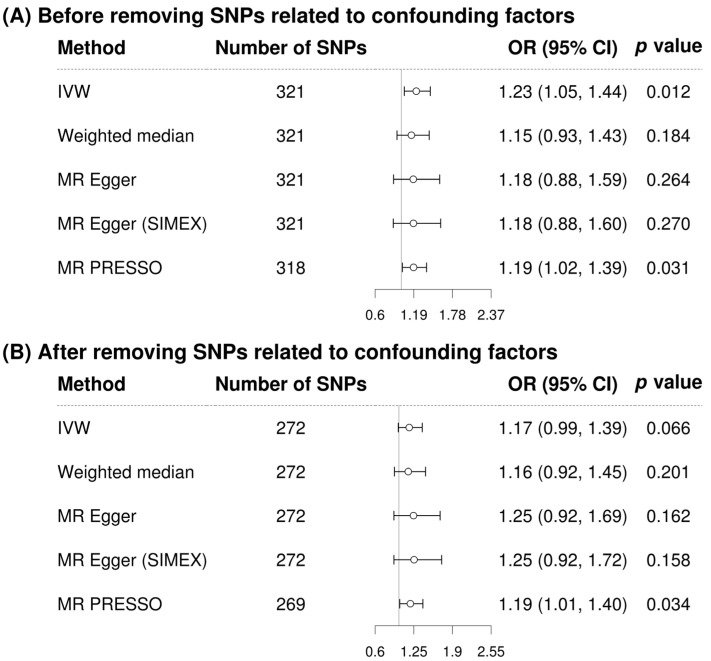
Forest plot of causal associations between gamma-glutamyl transferase and stroke. (**A**) Before removing SNPs related to confounding factors; (**B**) After removing SNPs related to confounding factors. Alcohol consumption, atrial fibrillation, and body mass index were considered as confounding factors. MR, Mendelian randomization; SNP, single-nucleotide polymorphism; IVW, inverse-variance weighted; SIMEX, simulation extrapolation; MR–PRESSO, MR-pleiotropy residual sum and outlier test; OR, odds ratio; CI, confidence interval.

**Figure 3:**
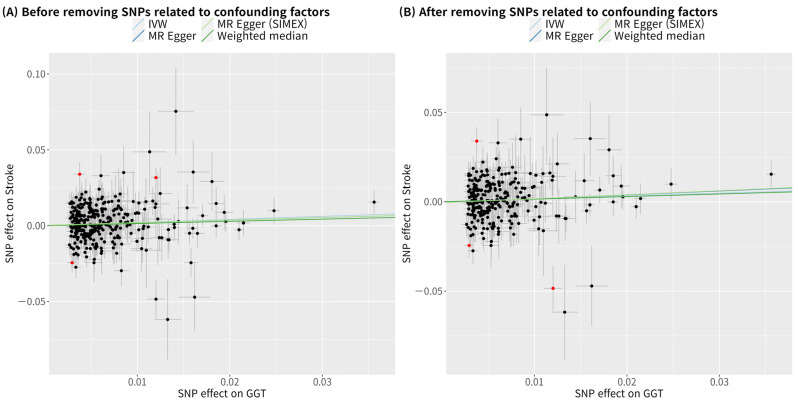
Scatterplots of MR tests assessing the effects of gamma-glutamyl transferase on stroke. (**A**) Before removing SNPs related to confounding factors; (**B**) After removing SNPs related to confounding factors. Alcohol consumption, atrial fibrillation, and body mass index were considered as confounding factors. Light blue, dark blue, light green, and dark green regression lines represent the IVW, MR–Egger, MR–Egger (SIMEX), and weighted median estimates, respectively. Red dots indicate outliers found in the MR–PRESSO analysis. SNP, single-nucleotide polymorphism; GGT, gamma-glutamyl transferase; IVW, inverse-variance weighted; SIMEX, simulation extrapolation; MR, Mendelian randomization; MR–PRESSO, MR-pleiotropy residual sum and outlier test.

In the single-variable conventional MR analysis, GGT level had a significant causal association with stroke when the analysis used 321 SNPs (IVW MR OR = 1.23, 95% CI: 1.05–1.44, *p* = 0.012; MR-PRESSO OR = 1.19, 95% CI: 1.02–1.39, *p* = 0.031, three SNPs excluded) (Figure 2). The MR results after removing confounding factor-related SNPs were similar (MR-PRESSO OR = 1.19, 95% CI: 1.01–1.40, *p* = 0.034, with 269 SNPs) (Figure 2). The scatterplot (Figure 3) also indicates that the risk of stroke increases with the GGT level. In the multivariable MR analysis, the relationship between GGT and stroke was still significant after adjustment of alcohol consumption, atrial fibrillation, and body mass index (OR = 1.30, 95% CI: 1.07–1.60, *p* = 0.010) (Table 3).

### 3.3. Mendelian Randomization for the Causal Association between Gamma-Glutamyl Transferase and Stroke Subtype

**Figure 4:**
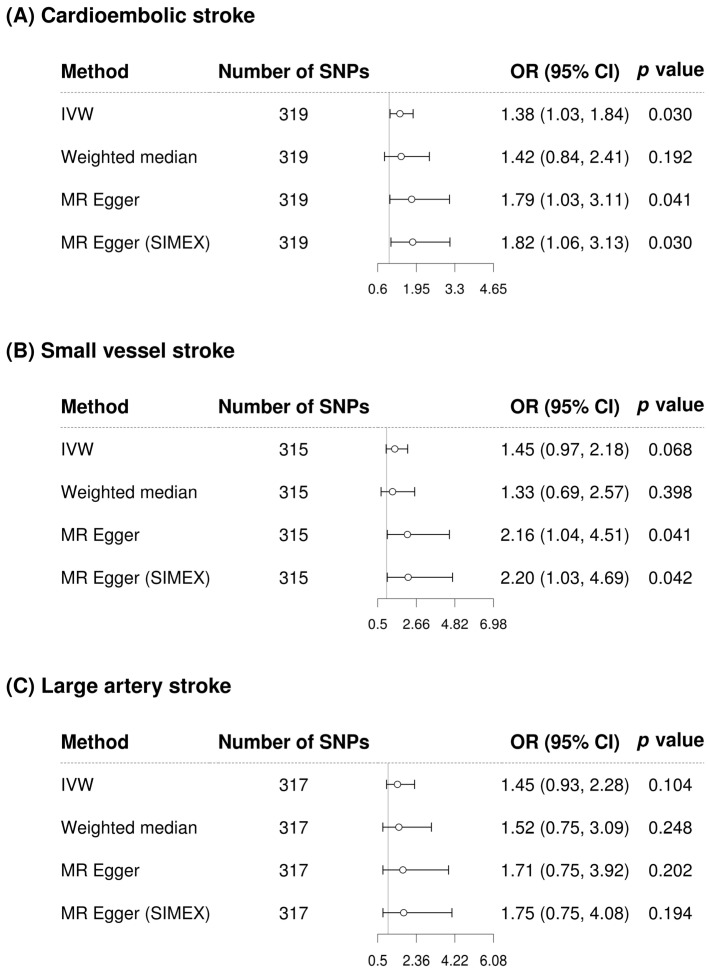
Forest plot of causal associations between gamma-glutamyl transferase and stroke-subtypes before removing SNPs related to confounding factors. (**A**) Cardioembolic stroke; (**B**) Small vessel stroke; (**C**) Large artery stroke. Alcohol consumption, atrial fibrillation, and body mass index were considered as confounding factors. SNP, single-nucleotide polymorphism; IVW, inverse-variance weighted; MR, Mendelian randomization; SIMEX, simulation extrapolation; OR, odds ratio; CI, confidence interval.

**Figure 5:**
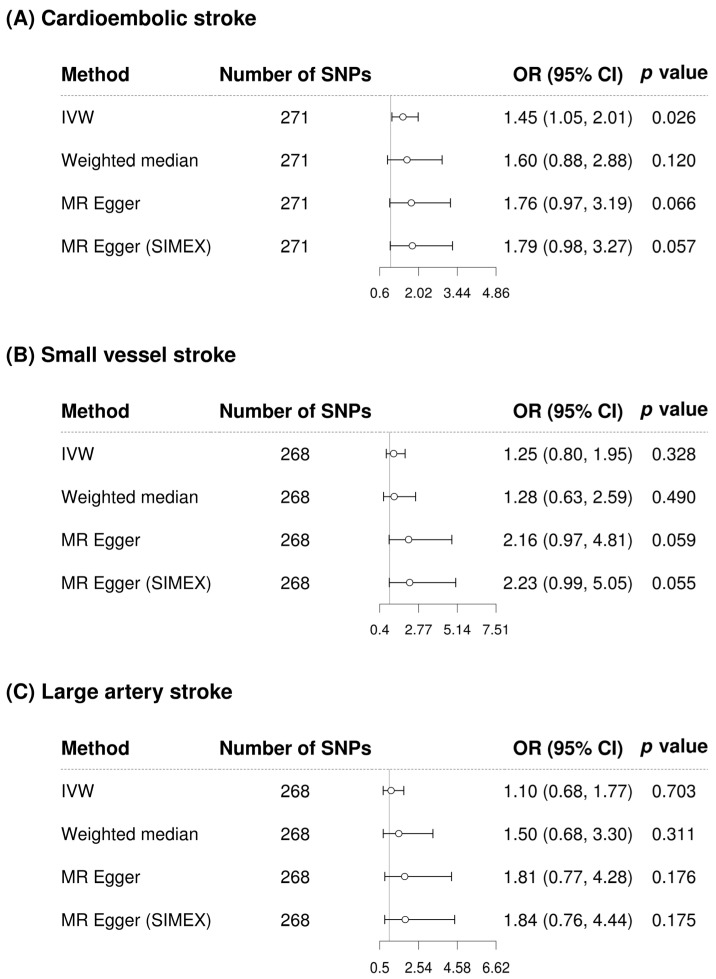
Forest plot of causal associations between gamma-glutamyl transferase and stroke subtypes after removing SNPs related to confounding factors. (**A**) Cardioembolic stroke; (**B**) Small vessel stroke; (**C**) Large artery stroke. Alcohol consumption, atrial fibrillation, and body mass index were considered as confounding factors. SNP, single-nucleotide polymorphism; IVW, inverse-variance weighted; MR, Mendelian randomization; SIMEX, simulation extrapolation; OR, odds ratio; CI, confidence interval.

**Figure 6:**
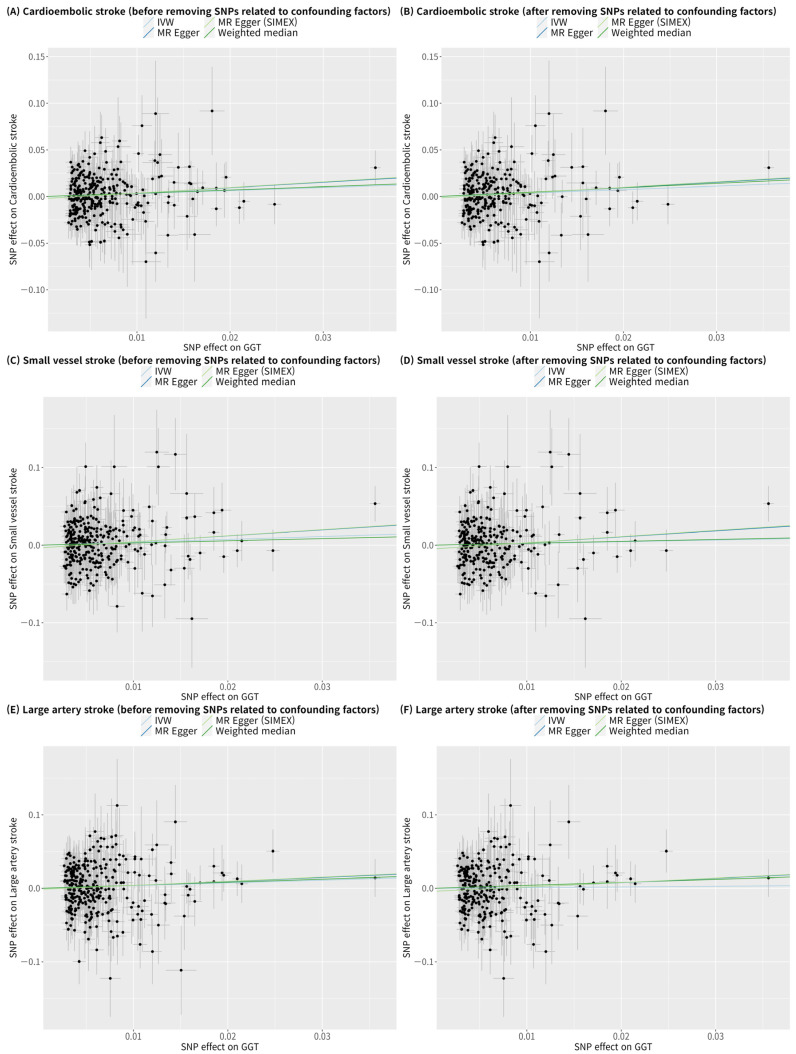
Scatterplots of MR tests assessing the effects of gamma-glutamyl transferase on stroke-subtypes. (**A**) Cardioembolic stroke before removing SNPs related to confounding factors; (**B**) Cardioembolic stroke after removing SNPs related to confounding factors; (**C**) Small vessel stroke before removing SNPs related to confounding factors; (**D**) Small vessel stroke before removing SNPs related to confounding factors; (**E**) Large artery stroke before removing SNPs related to confounding factors; (**F**) Large artery stroke before removing SNPs related to confounding factors. Alcohol consumption, atrial fibrillation, and body mass index were considered as confounding factors. Light blue, dark blue, light green, and dark green regression lines represent the IVW, MR–Egger, MR–Egger (SIMEX), and weighted median estimates, respectively. SNP, single-nucleotide polymorphism; GGT, gamma-glutamyl transferase; IVW, inverse-variance weighted; SIMEX, simulation extrapolation; MR, Mendelian randomization.

GGT appears to significantly increase the risk of cardioembolic stroke according to IVW in the single-variable conventional MR analysis regardless of whether confounding factor-related SNPs are removed (IVW MR OR = 1.38, 95% CI: 1.03–1.84, *p* = 0.030; IVW MR OR = 1.45, 95% CI: 1.05–2.01, *p* = 0.026 with 48 confounding factor-related SNPs excluded) (Figure 4 and Figure 5). However, after adjusting for alcohol consumption, atrial fibrillation, and body mass index, the relationship between GGT and cardioembolic stroke (OR = 1.15, 95% CI: 0.75–1.77, *p* = 0.531) was not significant in the multivariable MR analysis (Table 3). GGT was not significantly associated with small vessel stroke and large artery stroke according to IVW method in the single-variable conventional MR analysis regardless of whether confounding factor-related SNPs are removed (Figure 4 and Figure 5). Although MR-PRESSO was recommended for small vessel stroke (with or without removal of SNPs related to confounding factors) and large artery stroke (before removing SNPs related to confounding factors) but found no outliers. In multivariable MR analysis, GGT was significantly associated with small vessel stroke (OR = 1.79, 95% CI: 1.01–3.16, *p* = 0.046) and large artery stroke (OR = 1.94, 95% CI: 1.06–3.56, *p* = 0.032) after adjusting for alcohol consumption, atrial fibrillation, and body mass index. Scatterplots show the genetic association of GGT level against the genetic association with stroke subtypes for each SNP (Figure 6).

## 4. Discussion

Our study demonstrated a possible causal association between high GGT level and stroke. Moreover, GGT level demonstrated a causal association with stroke subtypes of cardioembolic stroke, small vessel stroke, and large artery stroke. Alcohol consumption has an impact on liver function tests, particularly the level of GGT [24]. Atrial fibrillation is reported to be linked to both stroke and GGT level [25]. Additionally, body mass index is a popular index for obesity, which is a risk factor for stroke [26], we performed a further analysis with the confounding factor-related SNPs (alcohol consumption, atrial fibrillation, and body mass index) removed, and the GGT level still had a causal effect on stroke. To address the limitations of the SNP elimination strategy, a multivariate MR analysis was conducted and revealed a significant causal association between GGT level and stroke subtype.

Serum GGT has been widely used as an index of liver dysfunction and a marker of alcohol intake. Conditions that increase serum GGT, such as obstructive liver disease, excessive alcohol use, and the use of enzyme-inducing medications, result in increased free-radical generation and the risk of glutathione depletion. Important advances have been made in defining associations between serum GGT and risk of coronary heart disease, type 2 diabetes, and stroke [44]. Several studies on the relationship between GGT and stroke have revealed GGT’s potential as a novel biomarker for stroke prediction [16,25,45,46,47]. Nevertheless, it remains unclear why GGT is associated with stroke. Several studies have suggested that GGT is associated with atrial fibrillation [25,48,49], and half of the association between GGT and cardioembolic stroke was mediated by atrial fibrillation. A mediation study revealed that the potential causal effect was mediated by GGT as well as atrial fibrillation [25]. Our MR analysis confirmed a causal relationship between GGT and stroke, as well as stroke subtype, but our inability to demonstrate a mechanism is a limitation. Nonetheless, it is a crucial discovery that GGT is an independent risk factor for stroke, as previously demonstrated by a large-scale study of 456,100 representative Koreans [33]. A recently meta-analysis on the association between GGT level and stroke risk showed that high GGT level was positively associated with increased risk of stroke (relative risk = 1.28; 95% CI, 1.61–1.43) [11], and that also supports our study. Our study has significant insights beyond those previous results because we demonstrated that GGT is a causal risk factor for stroke using European genetic data with the MR method. The observed variations in learning disabilities among racial groups present challenges in generalizing the findings of our study to other ethnic populations.

Stroke is widely recognized as a prominent contributor to both mortality and disability on a global scale [12]. The identification of individuals at risk of stroke at an early stage can effectively decrease the rates of mortality and morbidity associated with this condition. This early identification enables physicians to swiftly implement primary prevention methods. The primary modifiable risk factors are hypertension, diabetes mellitus, tobacco smoking, and hyperlipidemia, with lifestyle variables including obesity, inadequate diet/nutrition, and physical inactivity [50]. The enzyme GGT expedites the progression of atherosclerosis by means of oxidative and inflammatory processes. The potential link between GGT and atrial fibrillation has been postulated to be attributed to oxidative stress, chronic low-grade inflammation, and metabolic syndrome [51]. When an excessive amount of oxidative stress is present, there is a greater need for antioxidants, such as glutathione, to resolve it. In the present scenario, the necessity for GGT may also have been increased due to its role in the recycling of glutathione. In this regard, the examination of risk factors can encompass the influence of nutrition and inflammation. A recent study showed that diet was a substantial mitigating factor in the impact of obesity on the ratio of pro-inflammatory and anti-inflammatory interleukins, as well as on the levels of TGFβ-1 and GGT [52]. When examined through the lens of inflammation and oxidative stress, it may be inferred that there is a commonality between high GGT level and risk factors for stroke. Nevertheless, the absence of empirical support for a direct association between GGT and the progression of atrial fibrillation implies the necessity for an alternate hypothesis.

Understanding the causes of stroke is essential to creating successful preventive measures. The MR method uses genetic variations associated with a particular exposure to examine how changing that exposure affects disease risk. The advantages of MR research are that, when a set of genomic data and clinical information are available, the design is cross-sectional, but it can be rigorously verified by randomization, as in a prospective study [53,54]. In addition, unlike animal experiments, the MR method reduces the burden of random verification on humans and can demonstrate causality without putting participants at risk [55]. Several MR studies on stroke were previously performed with various results [19,56,57,58,59]. Smoking behavior was a causal factor for ischemic stroke in an MR analysis [20], and another MR study showed that smoking, body mass index, and waist/hip ratio were causally associated with stroke [21]. In the literature we reviewed, we found no MR analysis results about a causal association between GGT level and stroke. We did find studies that used MR analyses to link stroke with another indication of liver function, such as non-alcoholic fatty liver disease or bilirubin level [6,23,60,61,62]; in those studies non-alcoholic fatty liver disease had a limited effect on stroke and showed controversial results. Although we used two-sample MR as a research method to reduce bias, we deemed alcohol to be a confounding factor because it has a substantial effect on GGT level. Thus, we eliminated all drinking-associated SNPs. Several reports indicate that GGT level can serve as a biomarker for alcohol intake [63,64], and we deemed it necessary to eliminate and revaluate variables that could influence GGT level. Furthermore, the presence of atrial fibrillation was considered as a potential confounding variable due to its known impact on stroke incidence. Additionally, obesity was included in the analysis. However, because the results of a previous meta-analysis indicated that GGT is a risk factor for stroke independent of alcohol consumption [11], we suggest the reliability of our results prior to exclusion of SNPs related to alcohol. To address this issue, a multivariable MR analysis was conducted, revealing a noteworthy causal relationship with stroke subtype.

The chief strength of our study is our use of a relatively large cohort dataset in finding a possible causal association between GGT level and stroke. However, this study also has a few limitations. First, we did not have access to individual-level data, so we could not explore any potential nonlinear relationships or stratification effects. Second, the test procedures used to validate MR hypotheses do not provide complete validation. Violations of the MR assumptions can lead to invalid conclusions, so our results should be interpreted cautiously. Third, the summary GWAS data used in this study were collected from individuals of European ancestry, which may restrict the generalizability of our findings to non-European populations. Fourth, although the two-sample MR method requires the use of GWAS results from independent data, we were unable to obtain exposure and outcome results from completely independent sources because we used consortium data. The exposure data were obtained from GWAS of UKB samples, and the stroke GWAS, conducted using GIGASTROKE, includes UKB samples, resulting in substantial overlap between the exposure and outcome data. However, in previous research using the large-cohort MR analysis methodology [65], the IVW and weighted median results remained unaffected, and they influenced the bias of the MR-Egger results.

## 5. Conclusions

Our study using the MR method demonstrated a causal association between GGT and stroke and stroke subtype (small vessel stroke, and large artery stroke) in multivariable results. In addition, after removal of confounding factor-related SNPs, GGT continued to show a causal relationship with stroke. Considering the significance of GGT and liver function, researchers should further clarify and investigate the association between GGT level and stroke.

## Figures and Tables

**Table 1:** Data sources for the summary statistics.

Traits	Data Source	No. of Participants	Population	No. of Variants	Reference
Serum gamma-glutamyl transferase	UK Biobank (UKB)	437,194	European (U.K.)	16,901,631	[27]
Alcohol consumption	UKB	64,001 (31,460 cases + 32,541 controls)	European (U.K.)	11,831,323	[29]
Atrial fibrillation	The Nord-Trøndelag HealthStudy (HUNT), deCODE, the Michigan Genomics Initiative (MGI), DiscovEHR, UKB, and the AFGen Consortium	1,030,836 (60,620 cases + 970,216 controls)	European (U.S., Iceland, Norway, U.K.)	34,740,186	[30]
Body mass index	UKB	532,396	European (U.K.)	12,007,571	[31]
Stroke	The GIGASTROKE Consortium	2,036,031 (136,047 cases + 1,899,984 controls)	European (Iceland, Spain, Canada, Sweden, Netherlands, U.S., Finland, Denmark, U.K., France, Austrailia, Germany, Estonia)	7,511,476	[28]
Cardioembolic stroke		1,245,612 (10,804 cases + 1,234,808 controls)		6,659,793	
Small vessel stroke		1,241,619 (6811 cases + 1,234,808 controls)		5,974,028	
Large artery stroke		1,241,207 (6399 cases + 1,234,808 controls)		5,784,788	

**Table 2:** Heterogeneity and horizontal pleiotropy of the instrumental variables.

Outcome				Heterogeneity		Horizontal Pleiotropy				
				Cochran’s Q Test from IVW	Rucker’s Q’ Test from MR-Egger	MR-PRESSO Global Test	MR-Egger		MR-Egger (SIMEX)	
	N	F	I^2^ (%)	*p*-Value	*p*-Value	*p*-Value	Intercept, β (SE)	*p*-Value	Intercept, β (SE)	*p*-Value
Before removing SNPs related to confounding factors *										
Stroke	321	134.18	97.46	<0.001	<0.001	<0.001	0.000 (0.001)	0.764	0.000 (0.001)	0.776
Cardioembolic stroke	319	134.11	97.48	0.894	0.896	0.902	−0.002 (0.002)	0.272	−0.002 (0.002)	0.230
Small vessel stroke	315	134.82	97.50	0.038	0.041	0.035	−0.003 (0.003)	0.208	−0.003 (0.003)	0.202
Large artery stroke	317	134.27	97.51	0.006	0.005	0.005	−0.001 (0.003)	0.639	−0.002 (0.003)	0.614
After removing SNPs related to confounding factors *										
Stroke	272	131.77	97.48	<0.001	<0.001	<0.001	−0.001 (0.001)	0.650	−0.001 (0.001)	0.627
Cardioembolic stroke	271	131.36	97.50	0.681	0.674	0.683	−0.002 (0.002)	0.449	−0.002 (0.002)	0.414
Small vessel stroke	268	132.07	97.51	0.036	0.042	0.038	−0.005 (0.003)	0.106	−0.005 (0.003)	0.099
Large artery stroke	268	131.77	97.55	0.063	0.069	0.069	−0.004 (0.003)	0.171	−0.004 (0.003)	0.168

* Alcohol consumption, atrial fibrillation, and body mass index were considered as confounding factors. N, number of instruments; F, mean F statistic; IVW, inverse-variance weight; MR, Mendelian randomization; PRESSO, polyhedral sum of residuals and outliers; SIMEX, simulation extrapolation; β, beta coefficient; SE, standard error; SNP, single-nucleotide polymorphism.

**Table 3:** Multivariable IVW MR results of gamma-glutamyl transferase, alcohol consumption, atrial fibrillation, and body mass index on stroke and stroke subtypes.

	Stroke			Cardioembolic Stroke			Small Vessel Stroke			Large Artery Stroke		
MR Exposures	N	OR (95% CI)	*p*-Value	N	OR (95% CI)	*p*-Value	N	OR (95% CI)	*p*-Value	N	OR (95% CI)	*p*-Value
Serum gamma-glutamyl transferase	409	1.30 (1.07, 1.60)	0.010	397	1.15 (0.75, 1.77)	0.531	384	1.79 (1.01, 3.16)	0.046	384	1.94 (1.06, 3.56)	0.032
Alcohol consumption	409	0.96 (0.90, 1.03)	0.238	397	0.97 (0.84, 1.12)	0.665	384	0.88 (0.73, 1.07)	0.205	384	0.96 (0.78, 1.17)	0.663
Atrial fibrillation	409	1.13 (1.09, 1.17)	<0.001	397	1.86 (1.73, 2.01)	<0.001	384	1.02 (0.93, 1.13)	0.630	384	1.03 (0.92, 1.14)	0.616
Body mass index	409	1.10 (1.02, 1.18)	0.009	397	0.97 (0.83, 1.13)	0.677	384	1.16 (0.95, 1.41)	0.149	384	1.20 (0.97, 1.48)	0.100

N, number of instruments; IVW, inverse-variance weight; MR, Mendelian randomization; OR, odds ratio; CI, confidence interval.

## Data Availability

The datasets used and/or analyzed in the current study are available from the GWAS catalogue (https://www.ebi.ac.uk/gwas/summary-statistics, accessed on 7 July 2023). We used GWAS summary statistics from the following sources: GGT (study accession number GCST90013407), alcohol consumption (study accession number GCST90041731), atrial fibrillation (study accession number GCST006414), body mass index (study accession number GCST90029007), and stroke subtype GWAS generated by the GIGASTROKE consortium (study accession numbers GCST90104539, GCST90104541, GCST90104542, and GCST90104543).

## Supplementary Materials

The following supporting information can be downloaded at: https://www.mdpi.com/article/10.3390/biom13111592/s1, Supplementary Table S1: List of single-nucleotide polymorphisms used as instrumental variables in the single-variable MR analysis.
